# What Is the Role of HSCT in Philadelphia-Chromosome–Positive and Philadelphia-Chromosome–Like ALL in the Tyrosine Kinase Inhibitor Era?

**DOI:** 10.3389/fped.2021.807002

**Published:** 2022-02-02

**Authors:** Kim Vettenranta, Veronika Dobsinska, Gabriella Kertész, Peter Svec, Jochen Buechner, Kirk R. Schultz

**Affiliations:** ^1^University of Helsinki and Children's Hospital, University of Helsinki, Helsinki, Finland; ^2^Department of Pediatric Hematology and Oncology, National Institute of Children's Diseases, Comenius University, Bratislava, Slovakia; ^3^Department of Pediatric Hematology and Stem Cell Transplantation, Central Hospital of Southern Pest – National Institute of Hematology and Infectious Diseases, Budapest, Hungary; ^4^Department of Pediatric Hematology and Oncology, Oslo University Hospital, Oslo, Norway; ^5^Michael Cuccione Childhood Cancer Research Program, British Columbia Children's Hospital Research Institute, University of British Columbia, Vancouver, BC, Canada

**Keywords:** haematopoietic stem cell transplantation, tyrosine kinase inhibitors, Philadelphia chromosome, acute lymphoblastic leukaemia, BCR-ABL-like ALL, graft-vs.-host disease, graft-vs.-leukaemia effect

## Abstract

Previously, the outcome of paediatric Philadelphia-chromosome–positive (Ph^+^) ALL treated with conventional chemotherapy alone was poor, necessitating the use of haematopoietic stem cell transplantation (HSCT) for the best outcomes. The recent addition of tyrosine kinase inhibitors (TKIs) alongside the chemotherapy regimens for Ph^+^ ALL has markedly improved outcomes, replacing the need for HSCT for lower risk patients. An additional poor prognosis group of Philadelphia-chromosome–like (Ph-like) ALL has also been identified. This group also can be targeted by TKIs in combination with chemotherapy, but the role of HSCT in this population is not clear. The impact of novel targeted immunotherapies (chimeric antigen receptor T cells and bispecific or drug-conjugated antibodies) has improved the outcome of patients, in combination with chemotherapy, and made the role of HSCT as the optimal curative therapy for Ph^+^ ALL and Ph-like ALL less clear. The prognosis of patients with Ph^+^ ALL and persistent minimal residual disease (MRD) at the end of consolidation despite TKI therapy or with additional genetic risk factors remains inferior when HSCT is not used. For such high-risk patients, HSCT using total-body-irradiation–containing conditioning is currently recommended. This review aims to provide an update on the current and future role of HSCT for Ph^+^ ALL and addresses key questions related to the management of these patients, including the role of HSCT in first complete remission, MRD evaluation and related actions post HSCT, TKI usage post HSCT, and the putative role of HSCT in Ph-like ALL.

## Introduction

**Figure 1:**
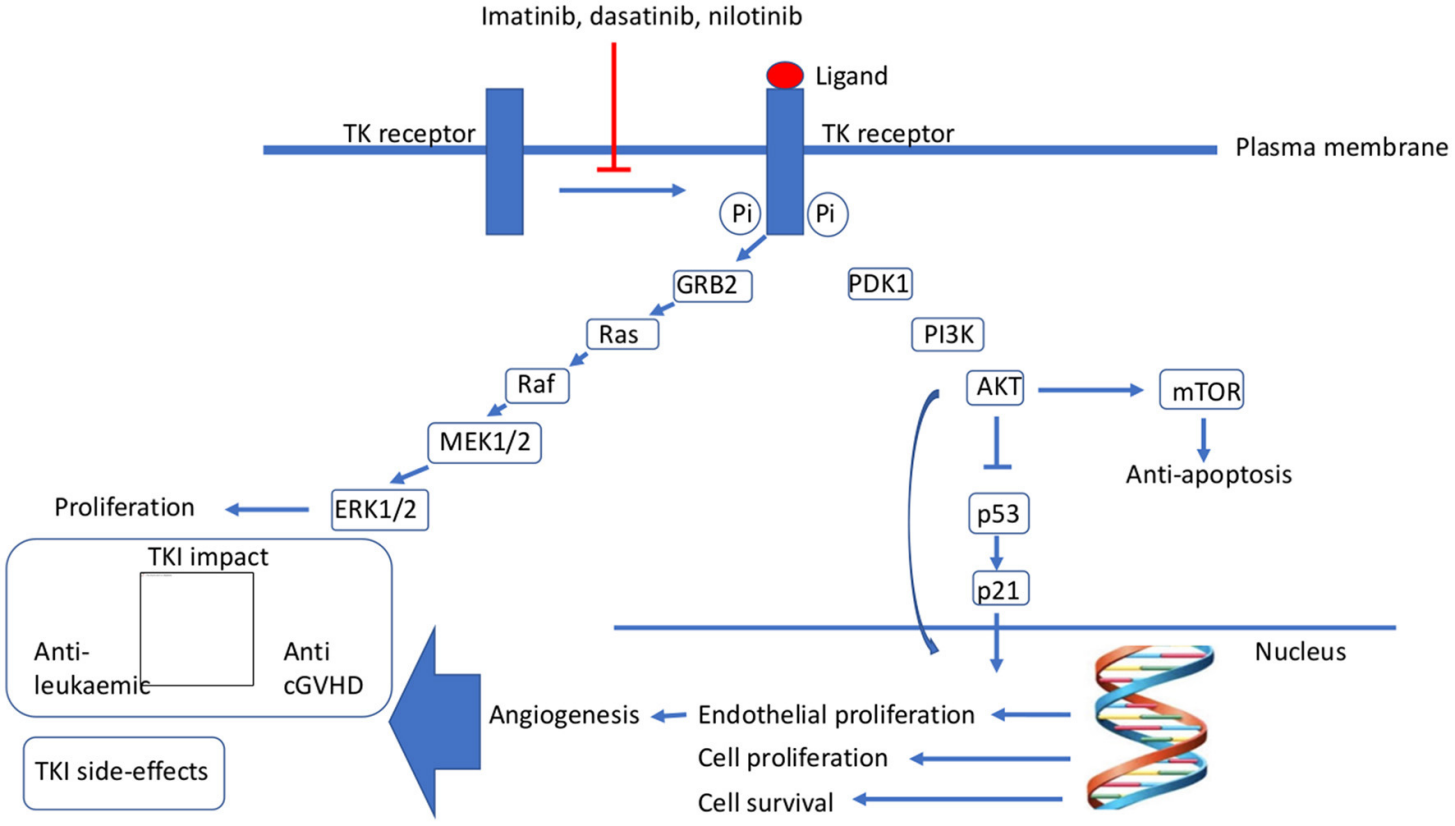
Mechanism of action of tyrosine kinase inhibitors. AKT, protein kinase B; cGvHD, chronic graft-vs.-host disease; ERK1/2, extracellular signal-regulated kinase 1/2; GRB2, growth factor receptor-bound protein 2; MEK1/2, mitogen-activated protein kinase 1/2; mTOR, mammalian target of rapamycin; Pi, phosphorylation; PI3K, phosphatidylinositol-3-kinase; TK, tyrosine kinase.

Philadelphia-chromosome–positive (Ph^+^) acute lymphoblastic leukaemia (ALL) and, more recently, also Philadelphia-chromosome–like (Ph-like; also known as *BCR-ABL*–like) ALL have been identified to be associated with poor prognosis when patients receive standard chemotherapy regimens (1–3). Ph^+^ ALL is found in fewer than 5% of paediatric patients with ALL but in more than 20% of adults with ALL, with the incidence in adolescents falling in between. With the advent of tyrosine kinase inhibitors (TKI) (Figure 1), the prognosis for paediatric patients with Ph^+^ ALL treated with TKIs added to the chemotherapy backbones began to approach that of non-Ph^+^ ALL patients (4–8). However, subgroups of Ph^+^ patients (e.g., those with *IKZF* mutations) with a substantially less favourable prognosis have been identified (6, 9). Allogeneic haematopoietic stem cell transplantation (HSCT) for consolidation of remission in Ph^+^ ALL patients is now reserved for those with specific high-risk features (2, 7). The role of HSCT in Ph-like ALL is less clear. In this review, we summarize the current role of HSCT in Ph^+^/Ph-like ALL.

## Recent Advances In The Chemotherapy Of Ph^+^ ALL And Their Impact On The Role Of HSCT

With the advent of TKIs, the role of HSCT in the treatment of paediatric Ph^+^ ALL has changed (summarized in Table 1) (4, 6, 8, 10–12). The non-randomized Children's Oncology Group (COG) AALL0031 trial added imatinib to an intensive chemotherapy backbone for the treatment of paediatric Ph^+^ ALL; only patients with a matched sibling donor (MSD) were stratified to undergo HSCT, with many patients taken off study for a matched unrelated donor (MUD) HSCT. No advantage of allogeneic HSCT was observed compared to the chemotherapy plus imatinib arm: 3-year event-free survival (EFS) was 87.7% for chemotherapy plus imatinib, 56.6% for MSD HSCT and 71.6% for MUD HSCT (5). The EsPhALL2004 trial, which also combined imatinib with chemotherapy for the treatment of paediatric Ph^+^ ALL, confirmed the outcome of the COG trial. In this trial, HSCT was indicated for poor-risk patients with any donor type and for good-risk patients with an MRD or MUD. When censored at the time of HSCT, the 2-year disease-free survival (DFS) was 81.2% in the good-risk group treated with imatinib vs. 65.4% in the good-risk group treated without imatinib. In the poor-risk group, in which 84% of patients underwent HSCT, the 4-year EFS was 53.5% (10). A third study in paediatric Ph^+^ ALL—EsPhALL2010—used a similar strategy to the AALL0031 study by giving imatinib continuously (300 mg/m^2^) but starting at an earlier timepoint of day 15 of the induction chemotherapy. Starting with the same HSCT indications as in the EsPhALL2004 protocol, the criteria were restricted in 2012 based on the consensus that good responders (defined by minimal residual disease [MRD] level at the end of consolidation) did not need HSCT. Thus, HSCT was reserved for the poor responders only (MRD ≥5 × 10^−^4). The 5-year overall survival (OS) for the group of patients undergoing HSCT in first complete remission (CR1) group was 77.3% compared to 73.6% for the non-transplanted patients (*p* = 0.63) (8).

**Table 1:** Summary of published trials investigating TKIs for Ph^+^ ALL in children and adolescents.

**Trial (reference)**	**Years**	**Patients, N**	**Chemotherapy**	**TKI**	**cXRT**	**HSCT**	**EFS**	**OS**
COG AALL0031 (4) NCT00022737	2002–2006	54	AALL0031	Imatinib 340 mg/m^2^	All	43%	5-yr: 68%	5-yr: 81%
EsPhALL (randomized) (10)	2004–2009	178	BFM HR	Imatinib 300 mg/m^2^	All	81%	5-yr: 60%	5-yr: 72%
COG AALL0622 (6) NCT00720109	2008–2012	60	AALL0031	Dasatinib 60 mg/m^2^	CNS3 only	32%	5-yr: 60%	5-yr: 86%
EsPhALL 2010 (8) NCT00287105	2010–2014	155	BFM HR	Imatinib 300 mg/m^2^	All	38%	5-yr: 57%	5-yr: 72%
CCCG-ALL-2015 (11) ChiCTR-IPR-14005706	2015–2018	92	Mod Total XV–XV1	Dasatinib (D) 80 mg/m^2^ vs. Imatinib (I) 300 mg/m^2^	None	4.3%	4-yr: 71% (D), 48.9% (I)	4-yr: 88.4% (D), 69.2% (I)
CA180-372 (12) NCT01460160	2012–2014	106	BFM HR	Dasatinib 60 mg/m^2^	CNS3 only	14%	5-yr: 54.6%	5-yr: 81.7%

*BFM, Berlin-Frankfurt-Münster; CCCG, Chinese Children's Cancer Group; COG, Children's Oncology Group; CNS3, central nervous system disease with a WBC count in the CSF ≥5 and blasts in the CSF; EsPhALL, European intergroup study of post-induction treatment of Philadelphia-chromosome-positive ALL; HR, high risk; TKI, tyrosine kinase inhibitor; cXRT, craniospinal radiotherapy; HSCT, haematopoietic stem cell transplantation; EFS, event-free survival; OS, overall survival; yr, year*.

Dasatinib in combination with chemotherapy was evaluated also in the COG AALL0622 trial of paediatric Ph^+^ ALL, with dasatinib added at day 15 to the identical chemotherapy backbone used in the AALL0031 trial. The 5-yr EFS was similar for the non-transplanted and transplanted groups (60 vs. 61%, respectively). The study concluded that HSCT should be limited to the high-risk group of slow responders as defined by the MRD levels. In addition, this trial suggested a potential role for transplantation in patients with additional IKZF1 deletions as a significant negative prognostic factor (6). In the COG AALL1122 phase 2 trial in paediatric Ph^+^ ALL, strategies from AALL0622 and EsPhALL2010 were merged and dasatinib (starting on day 15) administered with EsPhALL chemotherapy. The indication for HSCT in CR1 was restricted to patients with an MRD ≥0.05% at the end of consolidation or any MRD positivity following three additional high-risk chemotherapy blocks. An early study report showed that a substantially lower percentage of patients were transplanted in the trial compared to the percentages in the EsPhALL 2004 and 2010 trials, while similar outcomes were observed (5-year EFS was 54.6% in AALL1122 vs 60.3% in EsPhALL 2004 and 57% in EsPhALL 2010 for the whole pt cohort) (12).

With the possible benefit of dasatinib over imatinib remaining unestablished, the current EsPhALL2017/COG AALL1631 trial in paediatric Ph^+^ ALL (NCT03007147) was launched to study imatinib with randomization to EsPhALL (arm A) vs AALL1131-type chemo backbone using a non-inferiority design and imatinib in combination with the chemotherapy backbone. Only high-risk patients (MRD ≥5 × 10^4^ at end of consolidation) are being allocated to allogeneic HSCT. For the HSCT patients, the study is investigating the feasibility of administering imatinib post HSCT.

## HSCT in Ph^+^ ALL

With the success of the addition of a TKI to a chemotherapy backbone for the treatment of paediatric and adolescent Ph^+^ ALL, the future role of HSCT in the treatment of paediatric and adolescent Ph^+^ ALL remains to be delineated. HSCT represents a multimodal immune therapy for Ph^+^ ALL through a comprehensive immune response including T, B, natural killer (NK) and professional antigen-presenting cells. Ph^+^ ALL appears to respond well to immune therapy mediated by HSCT, with the overall survival rates hovering at 70–80% (8, 13) as compared with other subgroups of high-risk paediatric ALL such as hypodiploid ALL (8). Yet, HSCT is limited as a potential therapy primarily by its associated, immune-mediated toxicity as acute and chronic graft-vs.-host disease (GvHD) (Figure 1).

Currently, the majority of paediatric ALL patients undergoing HSCT receive TBI-containing conditioning (14), especially those with a very high relapse risk (15). TBI-based conditioning regimens are also widely used to prepare children with Ph^+^ ALL for HSCT (14). Due to the known late effects associated with the use of TBI (endocrine effects, reduced cognitive function, infertility, cataracts, and an increased risk of secondary malignancies), it has for a long time been a matter of intense debate whether chemoconditioning can effectively replace TBI. In their retrospective study, Friend et al. (14) found that ALL patients who received a non–TBI-based conditioning regimen had a lower 3-year EFS compared to those who received TBI (52 vs. 77%, respectively; *p* = 0.03). In their paper, but without a subgroup analysis, a small group of Ph^+^ patients were included, mostly in the non-TBI arm. Importantly, MRD positivity as measured by next-generation sequencing (NGS) prior to transplant was highly predictive of relapse: NGS-MRD negative patients had a 0% rate of relapse compared to a 50% relapse rate for the NGS-MRD–positive patients prior to HSCT (*p* = 0.04).

To further compare outcomes of TBI vs. chemoconditioning regimens, a multicentre European Society for Bone and Marrow Transplantation (EBMT) Paediatric Diseases Working Party (PDWP) retrospective study was performed. Paediatric patients with all subgroups of ALL (N = 3,054) transplanted between 2000 and 2012 were included. For children undergoing HSCT in CR1, the survival rates after TBI and chemoconditioning were not significantly different. For patients transplanted in CR2, the outcomes after TBI were superior to those after chemoconditioning with regard to leukaemia-free survival (LFS; 53.7 vs. 29.4%, respectively) and relapse incidence (30.6 vs. 49.3%, respectively) (16). The For Omitting Radiation Under Majority age (FORUM) trial—a large prospective international, randomized trial of HSCT in paediatric ALL—compared conditioning with TBI and etoposide to chemoconditioning regimens of busulfan or treosulfan in combination with fludarabine and thiotepa. This study found TBI-based conditioning to be associated with a significantly lower risk of relapse and treatment-related mortality (TRM) than either chemoconditioning regimen. In the Ph^+^ ALL group, TBI was superior to chemoconditioning with a 2-year EFS of 89 vs. 60%, respectively (13, 17). As a result, TBI prior to the HSCT is recommended for children ≥4 years of age with Ph^+^ ALL. However, TBI should be omitted in those of younger age (<4 years) due to its massive, toxic impact on the rapidly growing and developing child.

Optimal donor selection for HSCT in Ph^+^ ALL patients continues to be explored. An MSD is still the optimal donor but the optimal alternative donor source remains to be determined. Currently, the choices include unrelated umbilical cord blood, an MUD or mismatched unrelated donor (MMUD) or a haploidentical related donor. It has been suggested that umbilical cord blood may give a superior outcome compared to an unrelated adult donor (18) or at least a comparable outcome (19). At this time, it appears that all donor sources give similar results. One new approach has been the use of haploidentical HSCT to expand the donor availability, with strategies including *in vivo* T-cell depletion with post-transplant cyclophosphamide (PT-Cy) or *ex vivo* T-cell depletion (TCD) prior to HSCT.

*In vivo* depletion of the expanding, allo-reactive T cells with PT-Cy 48–72 h after transplant has been used in paediatric ALL of all subtypes (20, 21) with a reduction in both GvHD and graft rejection observed (22, 23). In adult ALL, there is no difference in the outcome between an MUD-HSCT and a PTCy haploidentical transplant (24), especially when using a TBI-containing conditioning regimen (25). The largest retrospective multicentre study on haploidentical HSCT to date analysed outcomes of 180 children with ALL after haploidentical HSCT using the PT-Cy modality (20). The estimated 2-year LFS was 65, 44, and 18.8% for patients transplanted in CR1, CR2, and CR3 or more, respectively, while 1-year LFS was 3% for those not in CR. The use of peripheral blood stem cells (PBSCs) was an independent factor associated with a decreased OS and higher NRM as opposed to bone marrow (20).

The other main approach to haploidentical HSCT is to perform *ex vivo* T-cell depletion prior to HSCT. Data on 343 patients with ALL who were <21 years old and who received their first allograft (αβ T-cell/B-cell depleted) after myeloablative conditioning in CR were analysed (26). The incidence of transplant-related complications was 6% with an MUD, 28% with an MMUD and 9% with a haploidentical graft. With a median follow-up of 3.3 years, the 5-year probability of LFS in the three groups was 67, 55, and 62%, respectively.

A review by Rahman and colleagues in the current *Frontiers in Pediatrics* supplement explores the different approaches to haploidentical HSCT in detail.

Currently, there is no evidence about which platform for haploidentical HSCT—PT-Cy or *ex vivo* T-cell depletion—is better, and no specific data on their use in Ph^+^/Ph-like ALL are available. A Spanish, multicentre, retrospective analysis of 192 children and adolescents with high-risk haematological malignancies compared the data of haploidentical HSCT using PT-Cy (*n* = 41) or *ex vivo* T-cell depletion (*n* = 151) in 10 centres between January 1999 and December 2016. The results of this study show that there are no statistical differences between the two approaches in terms of OS, DFS, GvHD-free, relapse-free survival, relapse, and TRM at day +100 (27).

## The Role of the Graft-vs.-Leukaemia Effect in Ph^+^ ALL

The GvL effect is closely associated with GvHD. To date, there is no identified immune target specific to Ph^+^ ALL that can be used to predict the GvL effect beyond general criteria used in ALL such as the human leukocyte antigen (HLA) DP (28). However, the gene fusion *BCR-ABL* itself has been targeted with tumour-specific T-cell therapy (29). In the COG ASCT0431 study, the presence of grade I–III acute GvHD (aGvHD) was associated with a lower risk of relapse of B-ALL (30). This association was confirmed by the FORUM trial showing that a moderately severe aGvHD (grade II) was associated with a GvL effect (17). While the GvL effect may be achieved without GvHD, milder forms of both aGvHD and chronic GvHD (cGvHD) appear to be associated with an augmented GvL effect, with a greater impact by aGvHD for paediatric ALL (31–33).

In a large retrospective CIBMTR study, researchers examined the GvL effect as a function of GvHD in both children and adults. Among the 5,215 transplant recipients, 1,619 were paediatric ALL patients in CR1/CR2 (with 15 % Ph^+^), and 1,003 had advanced disease (15% with Ph^+^). According to this study, GvHD was associated with an enhanced GvL effect in ALL. The beneficial effect of GvHD-associated GvL on the OS was confirmed for both the adults and children in CR1/CR2 with low-grade aGvHD (hazard ratio [HR], 0.49–0.69*)*, but not with cGvHD. In addition, a beneficial effect was shown in patients with advanced ALL and cGVHD with or without grade I or II aGvHD (reduction of mortality with HR, 0.83–0.76). The impact of pre-transplant MRD could not be evaluated as the MRD levels were unknown for 84% of patients (34).

A Japanese retrospective study on adult patients with Ph+ ALL failed to confirm the above CIBMTR study findings. The study evaluated 1,022 patients aged >15 years with Ph^+^ ALL who underwent HSCT to assess the impact of GvHD-associated GVL on the outcome of patients stratified by their MRD status. In contrast to the previous reports, the researchers did not observe a significantly better OS among those patients with a mild aGvHD or cGvHD regardless of MRD level (35).

What differentiates the treatment approach for Ph^+^ ALL from that used for other molecular subtypes of ALL is the addition of TKIs into induction therapy and, for some patients, also post-transplant. With use of post-transplant TKIs, their immunosuppressive effects become a consideration. The ability of TKIs to induce an immunomodulatory effect has been documented for T, NK and B cells. Also, regulatory T cell numbers are reduced among TKI-treated patients (36, 37). Pre-transplant TKIs increase the risk of infection, while post-transplant TKIs add to the immune suppression. The incidence and severity of cGvHD have been shown to be reduced by imatinib post HSCT (38).

## The Role of HSCT in Treating Ph-like ALL

A large subgroup of patients with a similar gene expression profile to Ph^+^ ALL without the classic *BCR-ABL* fusion gene (i.e., Ph-like ALL) were reported in 2009 as having a high rate of relapse with conventional chemotherapeutic regimens (39). Yet, the blast cells of these patients had rearrangements similar to Ph^+^ ALL such as *CRLF2* rearrangements, a *JAK* mutation or a variety of additional kinase alterations (*ABL1, JAK2, PDGFRB, EPOR, IL7R, SH2B3, FLT3* etc.). The resulting chimeric proteins showed substantial tyrosine kinase activity, even in the absence of high *ABL* expression (40).

The role of HSCT as a therapy for Ph-like paediatric ALL is uncertain (13). Childhood leukaemia study groups have focused on augmenting chemotherapy in combination with either Janus kinase 2 (JAK2) specific drugs, such as ruxolitinib, or ABL/platelet-derived growth factor receptor (PDGF-R) inhibitors, such as imatinib or dasatinib. Whether Ph-like ALL is as immunogenic and responsive to the HSCT-mediated GvL effect as Ph^+^ ALL needs to be determined. A retrospective evaluation through the CIBMTR or EBMT databases is needed to establish the efficacy of HSCT for Ph-like ALL. If it is similar to that in either infant *KMT2A*-rearranged or hypodiploid ALL, and thus relatively resistant to the GvL effect offered by HSCT, the outcomes with TKI therapy may not be as good as those seen for Ph^+^ ALL. While early results are promising, the ability of the targeted JAK2 or ABL/PDGF-R inhibitors to attain an MRD-negative state pre HSCT, putatively also of key importance in this, novel subgroup, remains to be established. The potential use of TKI therapy post HSCT in Ph-like also needs to be evaluated urgently. Either way, HSCT for Ph-like ALL is probably an important approach to offer as “total” immune therapy for this subpopulation of paediatric patients with ALL.

## The Role of MRD in HSCT for Ph^+^ and Ph-like ALL

Evaluation of treatment response in the form of sensitive MRD measurements in the post-induction period has been established as an indispensable tool for risk stratification in various ALL subtypes (41). The early European paediatric Ph^+^ ALL study, EsPhALL 2004, found that the achievement of MRD negativity after a consolidation phase resulted in a lower rate of relapse than that observed in patients with detectable MRD (5-year cumulative incidence of relapse: 14.3 vs. 35.3% respectively) (31). An end-of-consolidation MRD >5 × 10^−4^ or any MRD positivity at later timepoints stratifies patients into a high-risk arm to receive HSCT in CR1 in the current COG AALL1631/EsPhALL2017 trial. By contrast, the COG AALL0031 study using flow-cytometry–based MRD found that MRD positivity at the end of induction was not prognostic of outcome (4, 5).

In the next generation of international Ph^+^ ALL trials (AALL1631/EsPhALL2017 phase 3 trial), MRD measured by immunoglobulin (Ig) / T-cell receptor (TCR) real-time quantitative polymerase chain reaction (RQ-PCR) was selected as the primary method for measuring MRD (42). Although RQ-PCR quantification of genomic Ig/TCR and *BCR-ABL* RNA shows concordance (69% overall concordance in the EsPhALL2004 trial), *BCR-ABL* RQ-PCR remains more often positive at later timepoints, but without clear clinical significance, and appears to be less precise in predicting outcome (43). Use of *BCR-ABL* RQ-PCR was deemed impractical to measure MRD in the joint EsPhALL/ COG AALL1122 CA180-372 trial due to missing results caused by frequent, unmet assay requirements (44). The discordance between the Ig/TCR and *BCR-ABL* RQ-PCR results may be caused by the presence of *BCR-ABL1* translocation in non-leukaemic myeloid or other cells, possibly due to a CML-like stem cell disease (45).

**Figure 2:**
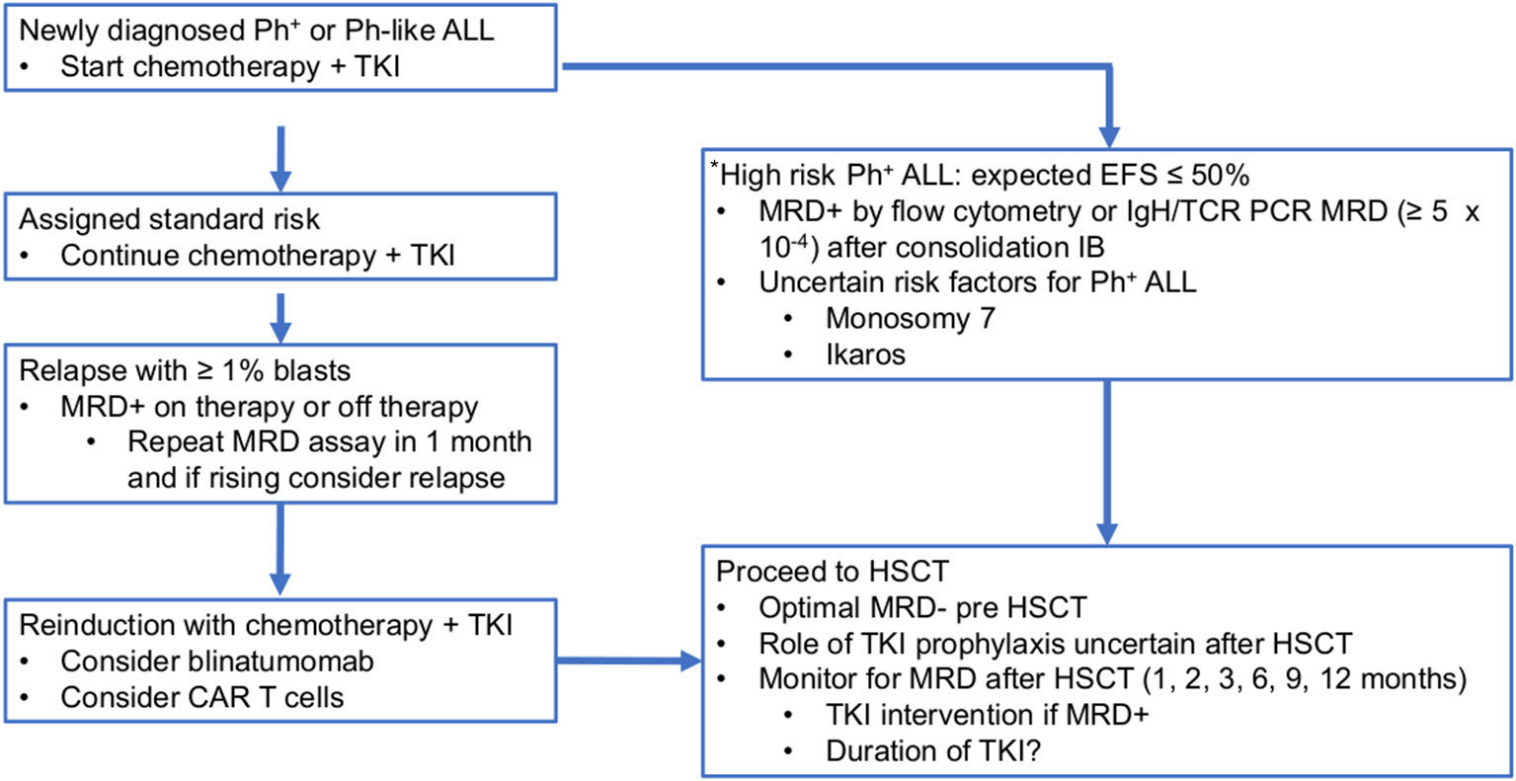
Proposed treatment algorithm for HSCT in paediatric Ph^+^ and Ph-like ALL. *There are no defined criteria for high-risk Ph-like ALL at present. ALL, acute lymphoblastic leukaemia; CAR, chimeric antigen receptor; HSCT, haematopoietic stem cell transplantation; IB, consolidation; MRD, minimal residual disease; Ph^+^, Philadelphia chromosome; TKI, tyrosine kinase inhibitor.

In the EsPhALL 2010 trial, nine (30%) of the 30 patients who were MRD negative at the end of consolidation and thus treated with imatinib plus chemotherapy relapsed vs. none of the 17 MRD-negative patients who underwent HSCT, similarly to EsPhALL 2004 (8). Thirty-three (37.8%) of the 87 MRD-negative patients treated with dasatinib plus chemotherapy in the EsPhALL/COG AALL1122 CA180-372 trial relapsed (44). This relapse rate of ≥30 % for the standard risk Ph^+^ ALL patients (MRD negative at the end of consolidation and no HSCT indication in CR1) suggests that the value of MRD negativity in Ph^+^ ALL for risk assessment is limited and differs from its role in the majority of the non-Ph^+^ ALL subtypes. Fortunately, a significant number of Ph^+^ ALL standard-risk patients can be salvaged after first relapse using TKI-containing chemotherapy regimens as bridging to HSCT (6, 8) (Figure 2).

Negative MRD pre HSCT, as well as concurrent aGvHD, is predictive of a lower rate of relapse in paediatric patients with ALL overall (46, 47). This appears to be true as well for Ph^+^ ALL (5). The relapse rate post HSCT for patients assigned to HSCT in the EsPhALL 2010 was five of 15 (33%) (8). In the EsPhALL/COG AALL1122 *CA180-372* trial it was four of 15 (44), a rate of relapse similar to other high-risk ALL patients undergoing HSCT for ALL in CR2. In the AALL0031 cohort, the 5-yr EFS rate for the MRD-negative patients after HSCT was 77% (5) and, interestingly, almost all patients in the EsPhALL2004 and EsPhALL/COG AALL1122 studies were Ig/TCR RQ-PCR negative or had low positivity before HSCT and had an excellent 5-yr EFS (86% in EsPhALL2004 and 91% in EsPhALL 2010). Thus, although MRD may not be as predictive for the outcome among patients receiving chemotherapy plus a TKI, it may be predictive for the HSCT outcomes (31). Moreover, the results of the AALL0622 study suggest that HSCT was able to abrogate the poor prognosis associated with MRD positivity at the end of consolidation (6).

The role of MRD monitoring for Ph^+^ ALL post HSCT is not well determined. The use of *BCR-ABL* PCR is uncertain and the results may come out as positive for a long time after HSCT and not predict relapse, at least not as previously described in adults (48). TCR-IgH PCR, flow cytometry or NGS are currently being utilized in several settings. While in CML peripheral blood *BCR-ABL* PCR correlates well with marrow measurements, evaluation of MRD in the marrow is still considered the standard for paediatric Ph^+^ ALL. Also uncertain is the optimal timing of MRD measurements after HSCT. Based on the high salvage rate of recurrent Ph+ ALL after chemotherapy plus TKI (6, 8), it is highly likely that patients who become MRD+ post HSCT will be reinduced into remission before a full relapse. In order to identify an early relapse post HSCT we recommend frequent monitoring of MRD after HSCT at 1, 2, 3, 6, 9, and 12 months after HSCT (c.f. Figure 2). These MRD evaluations may lead to a pre-emptive approach after HSCT although the level of MRD that should trigger the use of a TKI or another intervention is uncertain. Some experts have advised that a rise in MRD in measurements taken 2–4 weeks apart could be enough to launch a therapeutic intervention. Studies are needed to guide: (a) what method for the MRD measurement should be utilized; (b) what is an actionable “positive” MRD level; and (c) whether a TKI or other intervention is best.

Another unanswered question is for how long TKIs should be used pre-emptively in patients with MRD positivity. One year of treatment if MRD negativity is achieved is reasonable, with a close monitoring of the MRD once the TKI is discontinued. Another factor to consider is the impact of TKIs on haematopoiesis and immune responses, i.e., early TKI administration post HSCT (48) may require a lower dose than is standard. We expect that most clinicians would recommend imatinib as the preferred TKI to be used in a post-HSCT MRD-positive setting because it is the least marrow suppressive.

The role of HSCT for patients with BCR-ABL-like ALL is currently not known. Studies have been limited by data on the patients having a BCR-ABL-like translocation only recently being included in the data captured by the large HSCT databases of the CIBMTR and EBMT. Moreover, data on alternative immune therapies such as blinatumomab or CAR-T cell is only now being collected. It is reasonable to conclude that allogeneic HSCT is an excellent option for recurrent or refractory BCR-ABL-like ALL. Only through prospective clinical trials and retrospective analyses of the CIBMTR and EBMT databases with enough data will the relative efficacy of HSCT for this subtype of ALL be determined.

## The Impact of TKIs Post HSCT in Ph^+^ HSCT

The post-HSCT use of TKIs in both adult and paediatric Ph^+^ ALL has not been studied in a controlled way. In an EBMT retrospective study in adult with Ph^+^ ALL, a multivariate analysis found prophylactic TKI to significantly improve the LFS (hazard ratio, 0.44; *p* = 0.002) and lower the relapse incidence (hazard ratio, 0.40; *p* = 0.01) (49). On the other hand, the only randomized trial of post-transplant TKI reported that prophylactic and pre-emptive use of imatinib is equally effective in preventing relapse after allogeneic HSCT (50). A recent systematic review of 17 trials showed that the use of TKIs after allogeneic HSCT for patients in CR1 improved the OS when given either as a prophylactic or pre-emptive regimen but were of no benefit in patients with Ph^+^ ALL in CR2 or higher (51). Similarly, a retrospective analysis on 850 adult patients by the Japan Society for HSCT concluded that TKI prophylaxis was not associated with a decreased risk of relapse or superior OS in either MRD-negative or -positive patients in CR1 at HSCT (52). Also of importance are the immunosuppressive effects of TKIs, as demonstrated by imatinib's efficacy as a salvage treatment for steroid refractory cGvHD (53). While the EBMT retrospective study found a lower incidence of relapse only with aGvHD (49), a smaller retrospective study found that post-HSCT TKI prophylaxis was associated with a reduction in cGvHD (38). As opposed to the adult studies described above, no studies have evaluated the impact of post-HSCT prophylaxis or pre-emptive therapy on relapse and GvHD in children.

**Figure 3:**
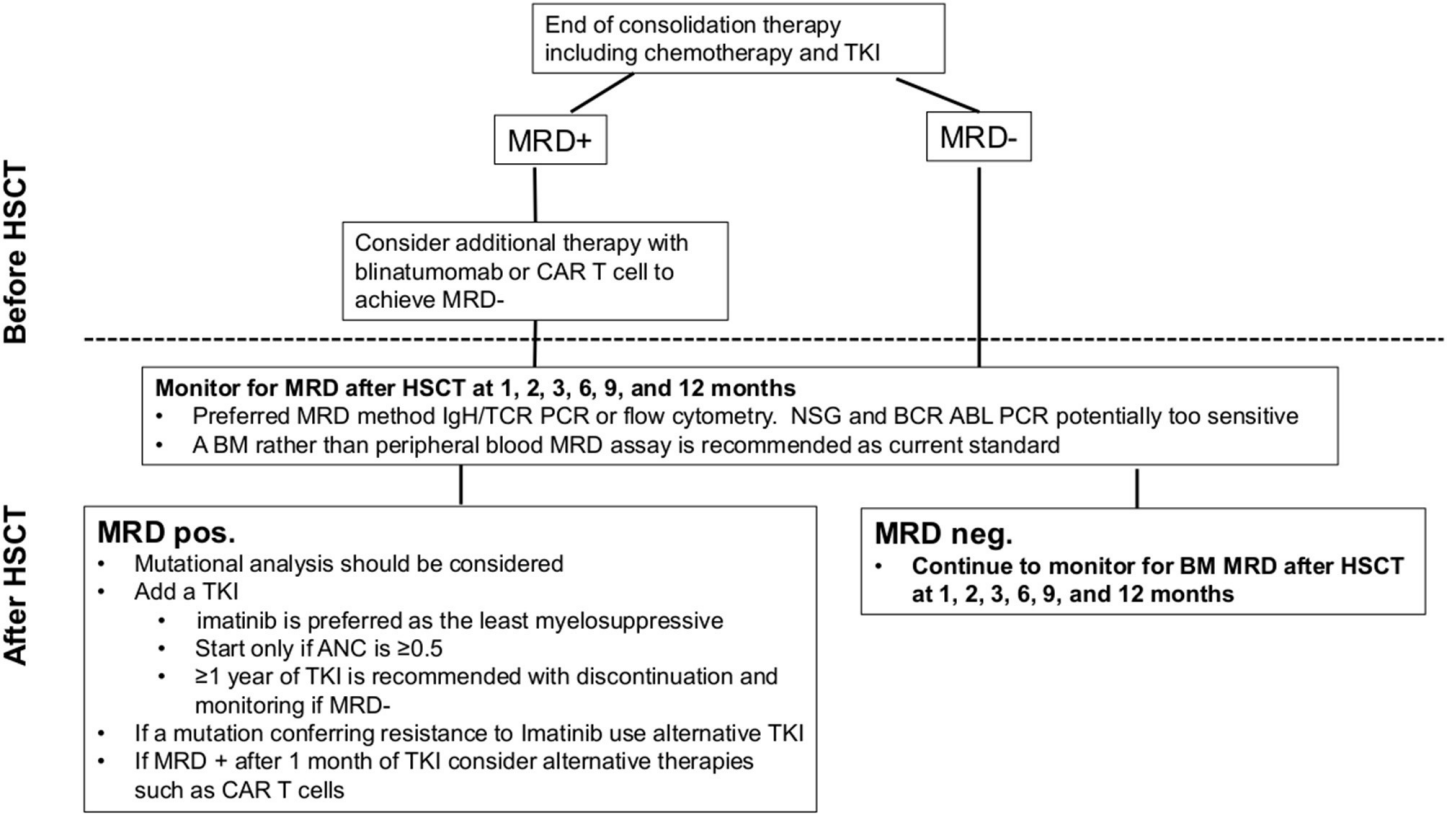
Our recommendations for the use of MRD to plan HSCT in paediatric Ph^+^ and Ph-like ALL. ANC, absolute neutrophil count; ALL, acute lymphoblastic leukaemia; BM, bone marrow; CAR, chimeric antigen receptor; HSCT, haematopoietic stem cell transplantation; IgH, immunoglobulin H; MRD, minimal residual disease; NGS, next-generation sequencing; PCR, polymerase chain reaction; Ph^+^, Philadelphia chromosome; TCR, T cell receptor; TKI, tyrosine kinase inhibitor.

When the TKIs are administered post HSCT, the optimal type, timing and duration remain to be decided for both the adult and paediatric patients. Limited data support the use of the newer generation TKIs for patients after HSCT (50, 51, 54). Examination of the mutational status and amplification of the *ABL* kinase gene is recommended in relapsed and non-responding patients. The initiation of TKI post HSCT requires a stable graft function to tolerate the myelosuppressive effect of the TKIs, usually seen from 2 months after HSCT (54). A reasonable duration of the TKI treatment is 6 months to 1 year of MRD negativity (51, 54). A retrospective analysis on the stopping of the TKIs post HSCT found that administration for more than 6 months tended to be associated with a superior relapse-free survival (55). Stopping TKIs post HSCT is often not a planned decision, as illustrated by a single prospective randomized trial on prophylactic vs. pre-emptive TKI post HSCT, where most patients in each group (67 and 71%) discontinued the treatment prematurely (48). Since the outcomes are similar for prophylactic vs. pre-emptive TKI therapy, the less-toxic pre-emptive strategy appears to be favourable but a strict MRD monitoring schedule needs to be implemented. The decision to use pre-emptive TKI therapy may be guided by an assessment of the pre- and post-HSCT relapse risk (51, 54). In conclusion, currently available data do not support the use of prophylactic TKI post HSCT. We recommend a pre-emptive approach based on the post-HSCT MRD analysis for those Ph^+^ ALL patients who are MRD negative at transplant (Figure 3).

## Approaches to Persistent MRD Positivity After HSCT in Ph^+^ and Ph-like ALL

One of the biggest challenges for the HSCT physician is when a patient remains MRD positive post HSCT after a TKI is implemented. Other targeted agents may become more commonly used, especially when MRD positivity persists after the implementation of a TKI and there is no mutation to suggest TKI resistance.

## The Role of Novel Immunotherapeutic Approaches in HSCT for Ph^+^ ALL

There are a number of targeted immune therapies that putatively will impact the role of HSCT in the treatment for high-risk Ph^+^ or Ph-like ALL in the paediatric population. One of the big questions to be answered is: “can HSCT be used to further improve outcomes in patients receiving a novel agent or can the novel immune therapies be used after HSCT to improve the outcome?”. The use of CAR T cells, blinatumomab or inotuzumab ozogamicin either to induce MRD negativity pre HSCT or as post HSCT prophylaxis or pre-emptive therapy remains to be elucidated. Their use in combination with TKIs may lead to novel approaches to achieve lower toxicity and higher efficacy in combination with HSCT.

## Recommendations and Conclusions

Our recommendations for HSCT in paediatric patients with Ph^+^ and Ph-like ALL are shown in Table 2. In summary, HSCT continues to offer an important therapeutic option for r/r Ph^+^ ALL in children and adolescents. However, the role of HSCT in Ph-like ALL, if any, is not clear, and additional studies are needed to establish the role of HSCT in this high-risk subpopulation. The role of TKIs in combination with HSCT for Ph-like paediatric ALL also requires further study.

**Table 2:** Key recommendations for the use of HSCT in paediatric Ph^+^ and Ph-like ALL.

**Clinical issue**	**Recommendation**
The impact of MRD pre HSCT	Aim for the lowest MRD possible prior to HSCT
Chemotherapy alone or TBI plus chemotherapy for conditioning	Use TBI-containing regimens only for patients >4 years old
Pre-emptive TKIs post HSCT	Use TKIs pre-emptively when indicated by MRD positivity during follow-up
The method of MRD follow-up post HSCT	Use PCR for IgH/TCR rearrangement(s) or flow cytometry to assess MRD post HSCT
The duration of the pre-emptive TKIs post HSCT	Post-transplant, use pre-emptive TKIs with a goal of 1 year of undetectable MRD
Progression or recurrence of disease	If progression or recurrence of ALL occurs, mutational analysis should be performed to ensure cancer cell sensitivity to the selected TKI

Optimal outcomes of HSCT for Ph^+^ ALL require the use of conditioning regimens with the lowest possible toxicity to establish MRD negativity pre HSCT, but should include TBI. Outcomes are similar for all donor sources. A better GvL effect may be achieved if either a low-grade aGvHD or cGvHD occurs after HSCT.

Routine MRD measurement are needed after HSCT and probably best performed by PCR for the IgH/TCR rearrangements or NGS rather than *BCR-ABL PCR* testing (56). There are currently no established data to support the consistent use of prophylactic TKIs post HSCT and, consequently, a pre-emptive approach based on close MRD monitoring post HSCT is probably the less toxic approach.

## Author Contributions

All authors listed have made a substantial, direct, and intellectual contribution to the work and approved it for publication.

## Funding

This study received funding from the St. Anna Children's Cancer Research Institute, Vienna, Austria. The funders were not involved in the study design, collection, analysis, interpretation of data, the writing of this article, or the decision to submit it for publication.

## Conflict of Interest

The authors declare that the research was conducted in the absence of any commercial or financial relationships that could be construed as a potential conflict of interest.

## Publisher's Note

All claims expressed in this article are solely those of the authors and do not necessarily represent those of their affiliated organizations, or those of the publisher, the editors and the reviewers. Any product that may be evaluated in this article, or claim that may be made by its manufacturer, is not guaranteed or endorsed by the publisher.
